# Combining genotypic and phenotypic analyses on single mutant zebrafish larvae

**DOI:** 10.1016/j.mex.2018.03.002

**Published:** 2018-03-14

**Authors:** Barbara Dupret, Pamela Völkel, Pauline Follet, Xuefen Le Bourhis, Pierre-Olivier Angrand

**Affiliations:** a Inserm U908, Cell Plasticity & Cancer, Lille, France; b University of Lille, Lille, France; c CNRS, Lille, France; d FRABio, CNRS FR3688, Lille, France; e SIRIC ONCOLille, Lille, France

**Keywords:** Zebrafish, Genotyping, Protein extraction, Histone extraction, RNA extraction, Histology, Spinal cord regeneration

## Abstract

Zebrafish is a powerful animal model used to study vertebrate embryogenesis, organ development and diseases (Gut et al., 2017) [1]. The usefulness of the model was established as a result of various large forward genetic screens identifying mutants in almost every organ or cell type (Driever et al., 1996; Haffter et al., 1996) [[2], [3]]. More recently, the advent of genome editing methodologies, including TALENs (Sander et al., 2011) [4] and the CRISPR/Cas9 technology (Hwang et al., 2013) [5], led to an increase in the production of zebrafish mutants. A number of these mutations are homozygous lethal at the embryonic or larval stages preventing the generation of homozygous mutant zebrafish lines. Here, we present a method allowing both genotyping and phenotype analyses of mutant zebrafish larvae from heterozygous zebrafish incrosses. The procedure is based on the genotyping of the larval tail after transection, whereas phenotypic studies are performed on the anterior part of the zebrafish larvae.

• The method includes (i) a protocol for genotyping, (ii) protocols for paraffin embedding and histological analyses, (iii) protocols for protein and histone extraction and characterization by Western blot, (iv) protocols for RNA extraction and characterization by RT-PCR, and (v) protocols to study caudal spinal cord regeneration.
• The technique is optimized in order to be applied on single zebrafish embryos and larvae.

## Graphical abstract

**Figure:**
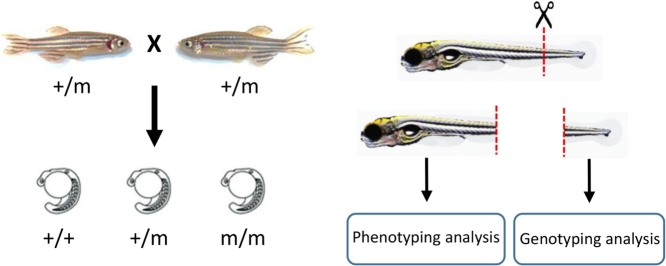

**Specifications Table**

**Table:** 

Subject area	• *Biochemistry, Genetics and Molecular Biology*
More specific subject area	*Zebrafish developmental biology*
Method name	Single zebrafish larvae characterization
Name and reference of original method	
Resource availability	

## Method details

Forwardand reverse genetics in zebrafish has established a huge variety of mutants [[1], [2], [3], [4], [5]]. Since numerous mutations are homozygous lethal at early developmental stages, methods allowing both genotyping and phenotype analyses of mutant zebrafish embryos and larvae are required. The method is designed to investigate the phenotype of sibling zebrafish larvae from heterozygous zebrafish incrosses. As so, single zebrafish larvae must be genotyped in order to eventually establish a correlation between different phenotypes and distinct genotypes. After transection, the larval tail is used for genotyping, whereas phenotypic studies are performed on the anterior part of the zebrafish larvae.

## Larval tail transection

### Equipment

• Sterile disposable scalpels (Paramount, # PDSS11)
• Stereomicroscope Leica M125 or equivalent
• Generic laboratory equipment

### Materials

• Living zebrafish embryos or larvae at the desired stage [between 5 and 12 days post-fertilization (dpf)]

### Reagents

• MS-222 (Sigma, # A5040)
• Trizma base (Sigma, # T1503)
• MS-222 stock solution: A 0.4% stock solution is prepared by dissolving 400 mg MS-222 in 97.9 mL H_2_O. The pH of the stock solution is adjusted to pH7–7.5 by adding 2.1 mL of Tris pH9, prepared by dissolving 260 mg Trizma Base into 2.1 mL H_2_O. This stock solution is stored at −20 °C.
• MS-222 anesthetic solution: 4.2 mL MS-222 stock solution are added to 100 mL tank water (final MS-222 concentration is about 0.016%).
• MS-222 overdose solution for euthanasia: 7.5 mL MS-222 stock solution are added to 92.5 mL tank water (final solution is about 300 mg/L)

### Procedure

1. Anesthetize zebrafish embryos or larvae with the MS-222 anesthetic solution in 6-well plates.
2. Sedated embryos or larvae are transferred using a P20 micropipettor equipped with a cut-off tip into a 10-cm Petri dish containing MS-222 anesthetic solution
3. Under a stereomicroscope, cut the tail with a sterile scalpel distal to the end of the intestine in order to preserve most of the embryo for phenotypic analyses and to recover a tail biopsy allowing easy handling and efficient DNA extraction (Fig. 1**A**).

**Fig. 1:**
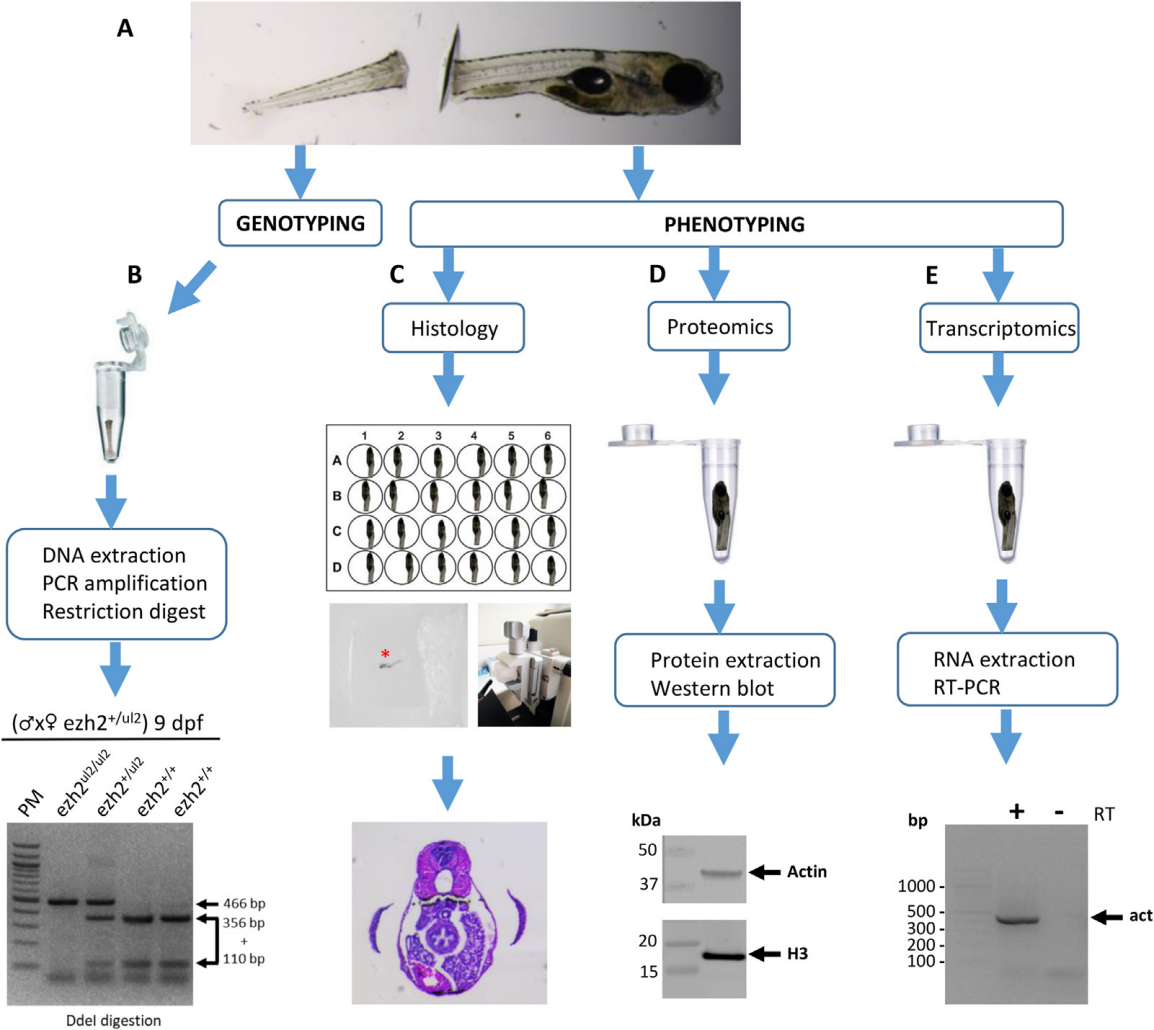
***Schematic representation of the strategy for the genotypic and phenotypic analysis on single zebrafish larvae.*** (**A**) Larval tail transection. (**B**) DNA is extracted from the tail biopsies and genotyping is performed by RFLP assay. (**C**) Histological study of the anterior part of the zebrafish larvae after paraffin embedding. The red asterisk shows the zebrafish larvae in the paraffin block. (**D**) Protein analysis by Western blot after total protein or histone extractions from the anterior part of a single zebrafish larvae. (E) Transcript analysis by RT-PCR after RNA extraction from the anterior part of a single zebrafish larvae.
4. Transfer the tail biopsy into a 0.2 mL sterile tube for DNA extraction whereas the anterior part of the larvae is transferred into a 24-well dish containing the MS-222 overdose solution for euthanasia and placed on ice

### Note

After 5–10 min in a MS-222 anesthetic solution bath, zebrafish embryos and larvae are fully sedated. Embryos and larvae may be kept in the anesthetic solution up to 2 h without effect on viability.

## DNA extraction from tail biopsies and genotyping

DNA extraction from zebrafish embryonic and larval tails is achieved using a protocol based on the use of sodium hydroxide and Tris, modified from Meeker et al. [6]. This method allows fast genomic DNA extraction used for genotyping by RFLP assay (Restriction Fragment Length Polymorphism; [7]). The method detects a mutation that either creates or abolishes a site recognized by a specific restriction enzyme. In the RFLP assay, a sequence of interest is first PCR-amplified and the PCR product is subjected to restriction enzyme digestion to identify whether the amplified alleles are wild-type or mutant.

### Equipment

• Thermocycler MJ Research PTC-200 or equivalent
• DNA electrophoresis tank and Power supply
• UV table equipped with a digital camera
• Generic laboratory equipment

### Reagents

• 50 mM NaOH
• 1 mM Tris-HCl, pH7.4
• Taq Polymerase (5U/μL), 10× Polymerase Buffer, 25 mM MgCl_2_ (Euromedex, # 01-01-02000)
• 10 mM dNTP Mix (Promega, # U1515)
• Forward and Reverse primers (10 μM), Eurogentec
• 10× Restriction Buffer
• Restriction Enzyme
• 50× TAE (Euromedex, # ET330)
• DNA loading buffer (New England Biolabs, # B70245)
• Agarose (Euromedex, # LE-8200-B)
• Ethidium bromide (Euromedex, # EU0071-A)

### DNA extraction procedure

1 Add 10 μL 50 mM NaOH to the tail biopsy
2 Heat the sample at 95 °C for 20 min before cooling it down at 4 °C for at least 15 min
3 Neutralize the solution by adding 1 μL 1 M Tris-HCl, pH7.4 (1/10 vol), vortex and centrifuge briefly.

### Genotyping

1 Transfer 2.5 μL of the lysate into a 0.2 mL PCR tube
2 Add 2 μL of 10× Polymerase buffer, 2 μL 25 mM MgCl_2_, 0.4 μL of 10 mM dNTPs, 1 μL of each forward and reverse primers (10 μM), 0.2 μL of Taq DNA polymerase (5 U/μL) and complete to 20 μL final volume with H_2_O.
3 Run the PCR as follows, 94 °C 2 min, [94 °C 20 s, 65 °C 45 s, 72 °C 30 s] 35 cycles, 72 °C 5 min
4 Digest 4 μL of the PCR product with 4 U of restriction enzyme and 1.5 μL 10× Restriction buffer in 15 μL final volume.
5 Incubate 3 h to overnight at the desired temperature.
6 Add 2 μL of DNA loading buffer to 15 μL digested sample and analyze on a 2% agarose electrophoresis gel in TAE.

Fig. 1**B** exemplifies the genotyping of the progeny of an *ezh2^ul2^* [8] heterozygous incross using this protocol. The *ezh2^ul2^* mutation removes a *Dde*I restriction site allowing genotyping by RFLP. PCR amplification of the *ezh2* wild-type gene using forward (5′-GGTATGGTTGTTGCAGTTCACAGAC-3′) and reverse (5′-AACACCAAACTCTACACAAGCAGCA-3′) primers generates a 466 bp DNA molecule which is cleaved by *Dde*I into 2 DNA fragments of approximately 356 bp and 110 bp. In contrast, PCR amplification and *Dde*I digestion of the *ezh2^ul2^* gene gives a 466 bp fragment resistant to *Dde*I cleavage. Under these conditions, it is possible to distinguish wild-type and *ezh2^ul2^* alleles by RFLP [8].

### Notes

• This procedure may also be used on paraformaldehyde-fixed samples to genotype entire embryos and larvae in 20 μL 50 mM NaOH after *in situ* hybridization, Alcian blue–Alizarin red staining or Oil red-O staining [[8], [9]].
• The setting of the PCR conditions may be modified according to the annealing temperature of the Forward and Reverse primers.
• In step 5, a master mix (N + 1) of all reagents could be made and 17.5 μL of the mix subsequently added to the DNA.
• In step 7, a master mix (N + 1) containing all restriction reagents may be used to ensure that all samples are equally proceeded.
• Since the identification of mutant alleles relies on the absence of a restriction site, including a wild-type control in the genotyping analysis may be a useful positive control.
• The protocol details a genotyping strategy based on RFLP, but alternate methods such as derived Cleaved Amplification Polymorphic Sequence (dCAPS; [10]) or High Resolution Melting Analysis (HRMA; [11]) genotyping assays could be used after the DNA extraction procedure.

## Histological analysis

For histological studies, sections of the anterior part of zebrafish embryos or larvae are performed after paraffin embedding.

### Equipment

• Glass pillboxes (Dutscher, # 211672)
• Hybridization oven
• Heating plate
• Stainless steel molds (Dutscher, # 040731)
• Paraffin fountain (Leica, # 1120)
• Microtome (Leica, # RM2245, or equivalent)
• Cover slips (WWR, # 631–1575)
• Glass slides, SuperFrost plus (Thermo, # 4951 PLUS4)
• Razor blades
• Generic laboratory equipment

### Reagents

• 4% paraformaldehyde (PFA) in Phosphate Buffer Saline (PBS) (wt/vol)
• Ethanol
• Claral (CML, RAL Diagnostic, # 320640-5000)
• Paraffin Paraplast Plus (Leica, # 39602004)
• Phosphate Buffer Saline (PBS)

### Procedure

1. Transfer the anterior part of the larvae into a 24-well dish after transection followed death in the MS-222 overdose solution (300 mg/L)
2. Remove as much as possible of the MS-222 solution and fix the samples by adding 1.5 mL 4% PFA. Embryos and larvae are fixed overnight at 4 °C
3. Wash the embryos or larvae once with PBS for 5 min
4. Transfer the embryos and larvae into glass pillboxes
5. Dehydrate the embryos and larvae with successive dilutions of ethanol in water: 10 min in 1.5 mL 30% (vol/vol) ethanol, 10 min in 1.5 mL 50% (vol/vol) ethanol, 10 min in 1.5 mL 70% (vol/vol) ethanol, 15 min in 1.5 mL 100% ethanol
6. Put the embryos and larvae for 10 min in 1.5 mL 50% ethanol – 50% Claral (vol/vol) at room temperature
7. Incubate embryos and larvae for 15 min in 1.5 mL 100% Claral at room temperature
8. After removal of the Claral, fill the glass pillboxes with pre-warmed paraffin in a paraffin fountain overnight at 58 °C, and incubate 15 min in an oven at 58 °C
9. Change the paraffin and incubate again for 15 min at 58 °C
10. Put the embryos and larvae in new warm paraffin solution into stainless steel molds and orientate the samples as required
11. Place the molds on the heating plate at 58 °C for 30 min. Then, on the heating plate at 37 °C for 1 h, and finally at room temperature for 4 h to overnight
12. Place the molds for at least 4 h at 4 °C before demolding and keep the zebrafish-containing paraffin blocks at 4 °C
13. Reduce the paraffin around the embryo with a razor blade
14. Stick the zebrafish-containing paraffin block to a bigger one (Fig. 1**C**) which is placed on the microtome
15. Perform 5 μm sections with the microtome
16. 16. Deposit the paraffin sections in a water drop onto a glass slide placed on a heating plate at 37 °C for 5 min and then remove the water
17. Place the glass slide on the heating plate at 37 °C for 30 min to 1 h maximum
18. Store the glass slides for at least 1 night at 37 °C in an incubator

Histological sections could then be analyzed by hematoxylin-eosin staining (Fig. 1**C**), TUNEL labeling, *in situ* hybridization, or *in situ* immunohistochemistry [8].

## Protein analysis by western blot

For protein studies (Fig. 1**D**), the anterior part of zebrafish embryos or larvae is transferred in a 1.5 mL Eppendorf tube, snap frozen in liquid nitrogen and kept at −80 °C for at least 1 night. Since epigenetic regulations, including histone modifications, play a crucial role in vertebrate development, the method describes both total protein and histone extraction procedures.

### Equipment

• Pestles for 1.5 mL tubes (Dutscher, # 045650)
• Cooling centrifuge for Eppendorf tubes (Eppendorf, # 5417R or equivalent)
• iBlot Dry Blotting System (Invitrogen, # IB1001W)
• Power supply
• Orbital shaker
• Hybridization oven
• Generic laboratory equipment

### Materials

• NuPAGE, 4–12% Bis-Tris polyacrylamide gel (Invitrogen, # WG1401BOX)
• Nitrocellulose membrane (Invitrogen, # IB3010-01)
• Parafilm M (Dutscher, # 90998)

### Reagents

• Triton X-100 (Sigma, # T9284)
• Tween 20 (Sigma, # P5927)
• NP40 (Sigma, # I3021)
• Phenyl-methylsulfonyl fluorid (PMSF) (Sigma, # P7626)
• NaN_3_ (Sigma, # S2002)
• Glycine (Sigma, # G7126)
• NaCl (Sigma, # S9888)
• 10× Phosphate Buffer Saline (PBS) (Euromedex, # ET330)
• 37% HCl (Sigma, # 320331)
• β-mercaptoethanol (Sigma, # M6250)
• DL-Dithiothreitol (DTT) (Sigma, # 43819)
• 20× MES SDS Running Buffer (Invitrogen, # NP0002)
• Milk powder (Regilait, skimmed milk)
• 2× Laemmli buffer (Sigma, # S3401)
• Triton Extraction Buffer (TEB): 0.5% Triton X-100, 2 mM PMSF, 0.02% NaN_3_ in PBS
• 4x Loading buffer (Invitrogen, NP0007)
• 1 M Tris-HCl pH6.8 (Genomic Solution, # 80-0161)
• 20× SDS (Biorad, # 161-0418)
• Primary and secondary antibodies
• PBST: 0.1% Tween 20 in PBS
• Blocking Buffer: 5% milk powder (wt/vol) in PBST
• Western Blotting detection System, Super Signal West Femto (Thermo, # 34096)
• 10× Reducing Agent is prepared by adding 1.6 mL β-mercaptoethanol and 3.78 g DTT in 10 mL H_2_O final. The solution is stored at −20 °C
• Stripping Buffer for total proteins: Add 4.5 mL 1 M Glycine, 9 mL 5 M NaCl, 450 μL 20% NP40 and 234 μL 37% HCl to 75.816 mL H_2_O (final volume is 90 mL)
• Stripping Buffer for histone blots: Add 3.125 mL 1 M Tris-HCl pH6.8, 5 mL 20% SDS and 350 μL β-mercaptoethanol to 41.525 mL H_2_O (final volume is 50 mL)

### Protein extraction procedure

1. Add 7.5 μL of 1× Laemmli buffer to the frozen embryo or larvae
2. Mash the embryo with a pestle in a 1.5 mL Eppendorf tube, at least 20 times
3. Add 1 vol (7.5 μL) of 1× Leammli buffer and mash the embryo again
4. Put the Eppendorf tube containing the mashed embryo 1 min in boiling water
5. Centrifugate the tube for 10 min at 20,800 g, 4 °C
6. Recover the supernatant and keep it at −80 °C for Western blotting

### Note

To study proteins of low abundance, several embryos or larvae from the same genotype could be pooled after freezing, in the same 1.5 mL Eppendorf tube. In this case, 15 μL of 1× Leammli buffer should be used for 5–10 embryos.

### Histone extraction procedure

1. Add 7.5 μL of Triton Extraction buffer (TEB) to the frozen embryo or larvae
2. Mash the embryo with a pestle in a 1.5 mL Eppendorf tube, at least 20 times
3. Keep on ice for 10 min
4. Centrifugate for 10 min at 20,800 g, 4 °C
5. Discard the supernatant and resuspend the pellet in 7.5 μL TEB with a P20 micropipettor
6. Centrifugate for 10 min at 20,800 g, 4 °C
7. Discard the supernatant and resuspend the pellet in 7.5 μL 0.2 N HCl with a P20 micropipettor
8. Extract the histones overnight at 4 °C on a rocking table
9. Centrifugate for 10 min at 20,800 g, 4 °C
10. Keep the supernatant containing the histone proteins at −20 °C
11. Add 3.75 μL 4× Loading buffer, 1.5 μL 10× Reducing Agent and adjust the volume to 15 μL with 0.2 N HCl

### Notes

• All the steps of the histone extraction procedure should be performed at 4 °C and the samples kept on ice.
• This method allows the recovery of approximately 3 μg histone proteins per 7 dpf embryo.
• If several embryos or larvae from the same genotype are processed together, mash 5–10 embryos in 15 μL TEB, resuspend in 7.5 μL TEB after the first centrifugation (step 5) and extract the histones with 7.5 μL 0.2 N HCl (step 7).
• Protein concentration is measured using the Bradford reagent (Biorad, # 500 00 06) with 2 μL of sample.

### Western blot procedure

1. Boil the samples 10 min at 95 °C
2. Load the protein samples onto a SDS denaturating gel (NuPAGE, 4–12% Bis-Tris polyacrylamide gel, Invitrogen) and run for about 90 min at constant 150 V
3. Transfer the proteins to a nitrocellulose membrane by iBlot Gel Transfer Stacks (Invitrogen) at 20 V for 7 min (preset program P3)
4. Block the membrane by incubation in Blocking Buffer, 1 h on an orbital shaker. Alternatively, membranes could be blocked overnight at 4 °C
5. Incubate the membrane with the primary antibody in Blocking Buffer for 1 h at room temperature. The incubation of the membrane with the antibody is performed by spreading the antibody solution with a pipette on the membrane, placed onto a Parafilm M flattened on the bench
6. Wash the membrane 3 times 10 min in PBST
7. Incubate the membrane with the secondary antibody in Blocking Buffer for 1 h at room temperature
8. Wash the membrane 3 times 10 min in PBST
9. Visualize the protein using a chemiluminescence substrate (Super Signal West Femto, Thermo) and a Luminescent Image Analyzer (LAS-4000, Fujifilm).

### Note

The Western blot results strongly rely on the quality of the antibodies. The limited number of zebrafish antibodies available often makes protein-based analyses more difficult.

Fig. 1**D** shows Western blots revealing the actin protein from total protein extracts and histone H3 from histone extraction from the anterior part of a single wild-type zebrafish larvae at 9 dpf. The primary antibodies used were rabbit anti-actin (1:10,000; A2066, Sigma) and rabbit anti-H3 (1:5000; ab1791, Abcam). The secondary antibody was a peroxidase conjugated donkey anti-rabbit antibody (1:10,000; 711-035-152, Jackson ImmunoResearch).

### Membrane stripping for re-use

1. Incubate the membrane 2 times 10 min at room temperature in freshly prepared Stripping Buffer for total proteins
2. Wash the membrane twice 5 min in PBST at room temperature
3. Block the membrane 1 h in Blocking Buffer at room temperature, before re-use with a Primary Antibody

For histone blots the procedure is modified as follow:

1. Incubate the membrane 30 min in an hybridization oven at 50 °C with pre-warmed Stripping Buffer for histone blots
2. Shake the membrane every 10 min
3. Wash the membrane 10 times 5 min in PBST at room temperature
4. Block the membrane 1 h in Blocking Buffer at room temperature before re-use with a Primary Antibody

## RNA analysis

For RNA studies (Fig. 1**E**), the anterior part of zebrafish embryos or larvae is transferred in a 1.5 mL Eppendorf tube, 200 μL Trizol is added before snap freezing in liquid nitrogen and storage at −80 °C for at least 1 night. The RNAs are extracted based on a modified version of the protocol from de Jong et al. [12].

### Equipment

• Pestles for 1.5 mL tubes (Dutscher, # 045650)
• Cooling centrifuge for Eppendorf tubes (Eppendorf, # 5417R or equivalent)
• Vortex
• Thermocycler MJ Research PTC-200 or equivalent
• DNA electrophoresis tank and Power supply
• UV table with digital camera
• Generic laboratory equipment

### Materials

• RNeasy Mini Kit (Qiagen, # 74104)
• SuperScriptIII Kit (Invitrogen, # 11752-050)

### Reagents

• Trizol (Ambion, # 15–596-018)
• Chloroform (Sigma, # 650498)
• Taq Polymerase (5 U/μL), 10× Polymerase Buffer, 25 mM MgCl_2_ (Euromedex, # 01-01-02000)
• 10 mM dNTP Mix (Promega, # U1515)
• Forward and Reverse primers (10 μM each), Eurogentec
• 100 bp DNA Marker (New England Biolabs, # N3231S)
• 6× Gel Loading Dye Purple (New England Biolabs, # B7024S)
• Agarose (Euromedex, # LE-800-B)
• 50× TAE (Euromedex, # ET330)
• Ethidium bromide (Euromedex, # EU0071-A)

### RNA extraction procedure

1. Put the zebrafish and Trizol-containg Eppendorf tubes on ice and mash the frozen samples with a pestle
2. Rinse the pestle with 100 μL Trizol to recover all material stuck to the pestle
3. Vortex 15 s
4. Keep 5 min at room temperature
5. Centrifugate for 15 s at 20,800 g
6. Add 60 μL chloroform (corresponding to 1/5 Vol. Trizol)
7. Vortex the sample for 15 s and then keep the tube 3 min at room temperature
8. Centrifugate for 20 min at 14,000 rpm (20,817 g), 4 °C
9. Transfer the upper aqueous phase (about 100 μL) in a new Eppendorf tube
10. Purify RNAs according to the RNA cleanup protocol provided in the RNeasy Mini Kit (Quiagen) following the manufacturer instructions and including the DNase digestion option
11. Recover the RNAs in 14 μL RNase free water

### Reverse transcription procedure

1. Transfer 7 μL of RNAs into a 0.2 mL PCR tube
2. Add 10 μL of RT Reaction Mix, 2 μL of RT Enzyme Mix and 1 μL of DEPC-treated water (final volume is 20 μL)
3. Mix gently and incubate for 10 min at 25 °C
4. Incubate for 30 min at 50 °C
5. Heat inactivate for 5 min at 85 °C and transfer the tube into ice
6. Add 1 μL RNase H
7. Incubate for 20 min at 37 °C
8. Store the cDNA samples at −20 °C

### Note

The Reverse transcription procedure follows the instruction provided with the SuperScriptIII Kit (Invitrogen).

### PCR procedure

1. Transfer 5 μL of cDNAs from the reverse transcription into a 0.2 mL PCR tube
2. Add 2.5 μL of 10× Polymerase Buffer, 0.5 μL 10 mM dNTPs Mix, 2.5 μL 25 mM MgCl_2_, 0.5 μL of each forward and reverse primers (10 μM), 0.5 μL Taq DNA Polymerase (5 U/μL) and 13 μL distilled water (final volume is 25 μL)
3. Run the PCR as follows, 95 °C 4 min, [95 °C 45 s, 55 °C 45 s, 72 °C 1 min] 35 cycles, 72 °C 10 min
4. Add 4 μL of Loading Buffer to the PCR product and analyze on a 1% agarose electrophoresis gel in TAE

### Notes

• The PCR conditions may be modified according to the annealing temperature of the Forward and Reverse primers.
• In step 2, a master mix (N + 1) of all reagents could be made and 20 μL of the mix subsequently added to the cDNA samples.

Fig. 1**E** shows the expression of actin revealed by RT-PCR from RNAs extracted from the anterior part of a single wild-type zebrafish larvae at 9 dpf. PCR amplification of the actin cDNA was performed using forward (5′-CGTGACATCAAGGAGAAGCT-3′) and reverse (5′-ATCCACATCTGCTGGAAGGT-3′) generates a 442 bp DNA fragment.

## Study of caudal spinal cord regeneration

The ability to regenerate tissues after amputation is part of the phenotypic studies that can be performed on zebrafish mutants at the larval stage [8].

### Equipment

• Sterile disposable scalpels (Paramount, # PDSS11)
• Stereomicroscope Leica M125 or equivalent
• Generic laboratory equipment

### Materials

• Living zebrafish embryos at 3 dpf

### Reagents

• MS-222 (Sigma, # A5040)
• Trizma base (Sigma, # T1503)
• MS-222 anesthetic solution:).
• 100× Penicillin-Streptomycin (Gibco, # 15140122)

### Procedure

1. Anesthetize zebrafish embryos at 3 dpf in MS-222 anesthetic solution in 6-well plates
2. Transfer the sedated embryos using a P20 micropipettor equipped with a cut-off tip, into a 10-cm Petri dish containing the MS-222 anesthetic solution
3. Under a stereomicroscope, cut the spinal cord with a sterile scalpel after the blood vessel within the pigment gap as described by Wilkinson et al. [13] and shown on Fig. 2**A**.

**Fig. 2:**
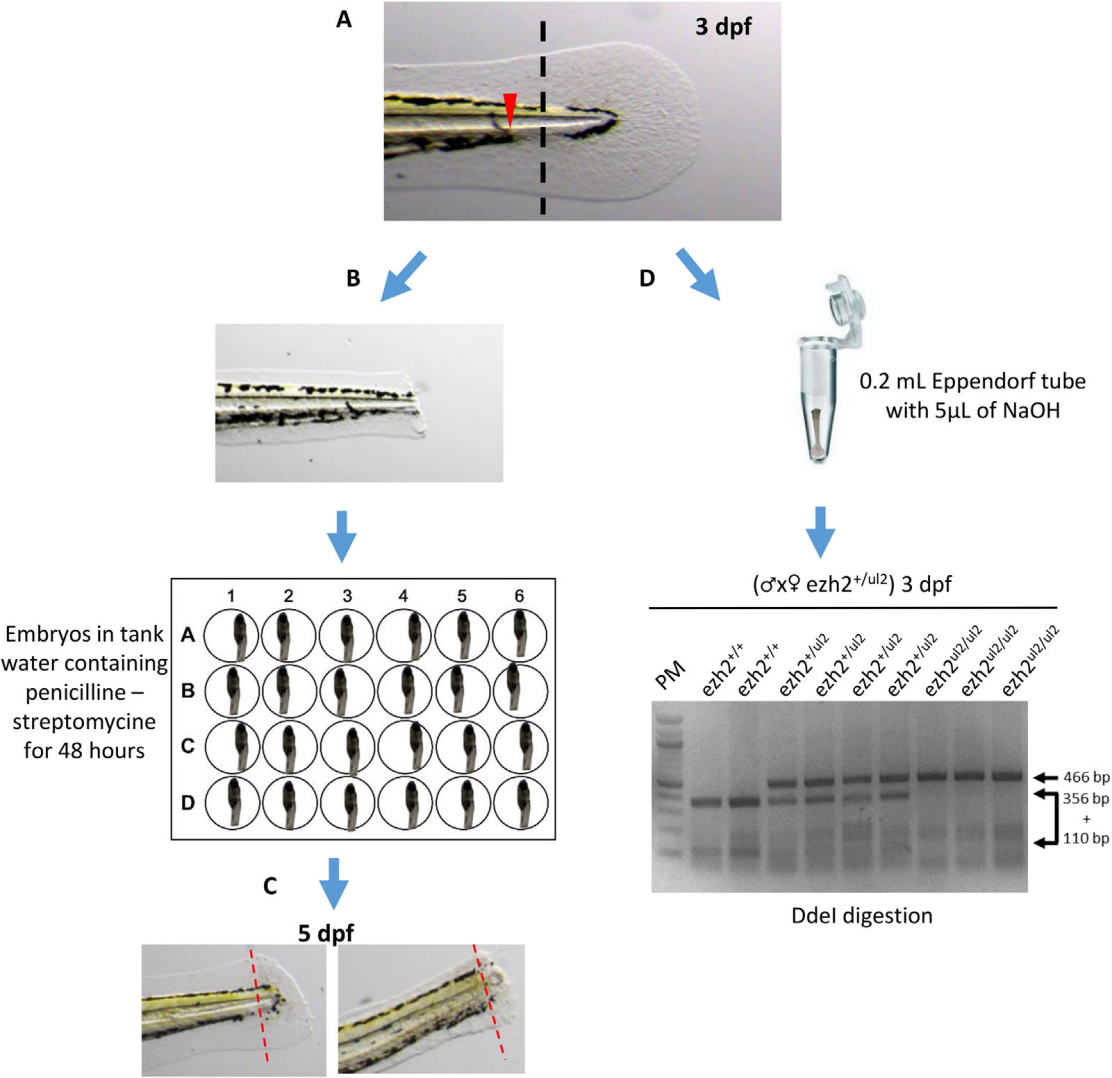
***Study of caudal spinal cord regeneration.*** (**A**) Caudal spinal cord transection on zebrafish embryo at 3 dpf. The dotted line represents the site of transection and the red arrowhead highlights the limit of blood circulation. (**B**) Picture of the caudal part of a transected zebrafish embryo at 3 dpf. (**C**) Embryonic caudal region of transected zebrafish embryos at 5 dpf, 2 days post-amputation, showing complete (left) or impaired (right) spinal cord regeneration. Red dotted lines indicate the site of transection. (**D**) Genotyping performed by RFLP on the caudal part of the embryos after transection at 3 dpf.
4. Transfer the tail biopsy for DNA extraction and genotyping as previously described
5. Transfer the embryos in a 24-well plate with tank water containing 1× Penicillin-Streptomycin (Fig. 2**B**)
6. Follow spinal cord regeneration 2 days after amputation (Fig. 2**C**)

Fig. 2**D** shows the genotyping of the progeny of an *ezh2^ul2^* [8] heterozygous incross using tail biopsy from 3 dpf embryos generated by this procedure.
