# Supplementary material for: Combining genotypic and phenotypic analyses on single mutant zebrafish larvae
Source: MethodsX. 2018 Mar 14;5:244–56. doi: 10.1016/j.mex.2018.03.002 (PMC6078847; doi:10.1016/j.mex.2018.03.002)
Supplement: Supplementary file 1 [file mmc1.docx]

**Supplementary material *and/or* Additional information:**

ANIMAL ETHICS STATEMENT. The zebrafish experiments described in this study were conducted according to the French and European Union guidelines for the handling of laboratory animals (Directive 2010/63/EU of the European Parliament and of the Council of 22 September 2010 on the protection of animals used for scientific purposes). At the end of the experiment, fish older than 6 dpf were humanely euthanized by immersion in an overdose of tricaine methane sulfonate (MS-222, 300 mg/L) for at least 10 minutes, whereas younger fish were immobilized by submersion in ice water (5 parts ice/1 part water, 0-4°C) for at least 1 hour to ensure death by hypoxia.
